# MAFF-Net: Multi-Attention Guided Feature Fusion Network for Change Detection in Remote Sensing Images

**DOI:** 10.3390/s22030888

**Published:** 2022-01-24

**Authors:** Jinming Ma, Gang Shi, Yanxiang Li, Ziyu Zhao

**Affiliations:** College of Information Science and Engineering, Xinjiang University, Urumqi 830046, China; majinming@stu.xju.edu.cn (J.M.); liyanxiang@stu.xju.edu.cn (Y.L.); 107551901060@stu.xju.edu.cn (Z.Z.)

**Keywords:** remote sensing images, change detection, attention mechanism, cross-layer feature fusion

## Abstract

One of the most important tasks in remote sensing image analysis is remote sensing image Change Detection (CD), and CD is the key to helping people obtain more accurate information about changes on the Earth’s surface. A Multi-Attention Guided Feature Fusion Network (MAFF-Net) for CD tasks has been designed. The network enhances feature extraction and feature fusion by building different blocks. First, a Feature Enhancement Module (FEM) is proposed. The FEM introduces Coordinate Attention (CA). The CA block embeds the position information into the channel attention to obtain the accurate position information and channel relationships of the remote sensing images. An updated feature map is obtained by using an element-wise summation of the input of the FEM and the output of the CA. The FEM enhances the feature representation in the network. Then, an attention-based Feature Fusion Module (FFM) is designed. It changes the previous idea of layer-by-layer fusion and chooses cross-layer aggregation. The FFM is to compensate for some semantic information missing as the number of layers increases. FFM plays an important role in the communication of feature maps at different scales. To further refine the feature representation, a Refinement Residual Block (RRB) is proposed. The RRB changes the number of channels of the aggregated features and uses convolutional blocks to further refine the feature representation. Compared with all compared methods, MAFF-Net improves the *F*1-Score scores by 4.9%, 3.2%, and 1.7% on three publicly available benchmark datasets, the CDD, LEVIR-CD, and WHU-CD datasets, respectively. The experimental results show that MAFF-Net achieves state-of-the-art (SOTA) CD performance on these three challenging datasets.

## 1. Introduction

Remote sensing image change detection (CD) uses two or more remote sensing images of the same area at different times to compare and analyze the atmospheric, spectral, and sensor information through artificial intelligence or mathematical statistics to obtain the change information of the area [1,2]. CD is an important research direction in the field of remote sensing and plays a great role in many fields such as land planning, urban expansion [3,4], environmental monitoring [5,6,7], and disaster assessment [8] as a key technology for monitoring surface conditions.

Recently, with the gradual maturity of remote sensing imaging technology, remote sensing image data with high resolution (HR) have been emerging. Compared with medium-resolution and low-resolution remote sensing images, HR remote sensing images have richer geometric and spatial information, which provide favorable conditions for humans to monitor surface changes more accurately. Therefore, the authors have paid more attention to the processing of HR remote sensing images. Effectively extracting the rich feature information of HR remote sensing images, better focusing on the change regions, avoiding the interference of other factors, and reducing the interference of pseudo-changes are the key issues of remote sensing image CD research [9].

There are many CD methods proposed, and different authors have made a more comprehensive summary classification from different aspects. In this paper, we will summarize and compare two perspectives from traditional methods and deep learning-based methods.

The traditional methods are divided into pixel-based remote sensing image CD methods and object-oriented remote sensing image CD methods according to the size of the basic unit [10]. The pixel-based remote sensing image CD method usually directly processes the input image according to the pixel-level spectral features, texture features, and other specific meaningful features (water bodies, vegetation indices). It obtains the difference image by difference or ratio. The change information is then extracted using a threshold segmentation method [11]. In the early days, methods such as image difference [12], image ratio [13], and regression analysis [14] were commonly used. However, these methods usually failed to obtain complete change information. To better utilize the spectral information of images, methods based on image transformation such as independent component analysis (ICA) [15] and multivariate alteration detection (MAD) [16,17] have emerged one after another and have achieved good results in land CD. For multispectral remote sensing images, the change vector analysis (CVA) [18] method is proposed to detect different changes in the ground. The CVA methods calculate the amplitude and phase angle and use the phase angle information to subdivide the changes. However, the performance of this type of method depends heavily on the quality of the spectral bands involved in the calculation, and the stability of the algorithm cannot be guaranteed. Therefore, improved versions of the CVA technique have been proposed during 2012–2016 to further improve the performance of CD [19,20,21,22]. With the development of HR optical remote sensing satellite technology, more and more HR remote sensing images are used for CD. 

The characteristic of “different objects in the same spectrum” in HR remote sensing images easily leads to the phenomenon of “salt and pepper” in the detection results. This problem further limits the practical application of pixel-level CD methods in HR remote sensing images [23]. Object-based CD methods are commonly used in HR remote sensing image CDs. This is because it allows for a richer representation of information. Ma et al. [24] investigated the effects of semantic strategy, scale, and feature space on an unsupervised, object-based CD method in urban areas. Subsequently, Zhang et al. [25] proposed an object-based CD method for unsupervised CD by incorporating a multi-scale uncertainty analysis. Zhang et al. [26] proposed a method based on the box-whisker plot with cosine law, which outperformed the traditional CD method. For CD tasks where “from–to” change information has to be determined, Gil-Yepes et al. [27] and Qin et al. [28] utilized a post-classification comparison strategy. Although the object-based CD method can better utilize the spatial feature information of HR remote sensing images compared with the pixel-based CD method, it also relies on the traditional manual feature extraction method, which is not only complicated and low-efficiency, but also has less stable CD performance [9]. In recent years, deep learning methods have been widely used in natural language processing, speech recognition [29,30], and image processing [31,32,33]. Deep learning methods have excellent learning ability and do not require the manual design of feature factors to extract features. With the success of deep learning in the field of image processing, deep learning-based CD for remote sensing images has quickly attracted the interest of scholars. With the continuous development of technology, the field of remote sensing CD has also started to make some excellent research based on convolutional neural networks (CNNs) [34]. CNNs do not require feature extraction by manually designed features. In the field of remote sensing CD, ResNet [35], full convolutional networks (FCN) [36], and UNet [37] structures have been widely used for feature map extraction with certain results. With continuous research, the model of remote sensing CD has been continuously optimized and improved.

For example, the FC-EF [38] network performs a concatenation operation before feeding two images into the backbone network of the UNet structure, then processes the images separately through two branches of the network. These two branches have the same network structure and shared parameters, and, finally, the outputs of the two branches are combined using convolutional layers. The FC-Siam-conc [38] and FC-Siam-diff [38] improve the network by jump-connecting the three feature maps from the two encoder branches and the corresponding decoder layer. FC-Siam-diff improves the network by first differencing the feature maps of the two decoder branches, then finding the absolute value of the difference, finally using a skip connection strategy to connect with the corresponding decoder layer. Subsequently, the FCN-based UNet network was successfully applied to the CD task [39,40], which was trained in an end-to-end manner from scratch using only available CD datasets. Coarse-to-fine [41] proposes a detection framework based on coarse-to-fine detection to detect remote sensing change regions. It firstly uses an encoder and decoder to obtain coarse change maps of bi-temporal images, then applies the idea of residuals to obtain refined change maps. The method can effectively detect the change regions with good results. After considering the feature maps between different layers with the idea of residuals, many scholars also use the attention mechanism in the direction of remote sensing CD to extract richer and finer feature maps. ResNet is used as a backbone by STANet [42], and then a self-attention module for CD is added in the process of feature extraction, which can calculate any two pixels. The authors of this model introduced Transformer on top of ResNet, which makes the network performance further improved [43]. DASNet [44] proposes a dual-attention mechanism to generate better feature representations to enhance the performance of the network. Zhang et al. [45] first use the two Siamese network architectures as the raw images feature extraction network. To enhance the integrity of change map boundaries and internal densities, multi-level depth features are fused with image difference map features by an attention mechanism. In 2021, Hou et al. [46] proposed a novel attention mechanism for mobile networks by embedding location information into channel attention, calling it Coordinate Attention (CA). CA enhances feature representation. In addition, in 2021, HDFNet [47] uses the idea of a hierarchical fusion and dynamic convolution model to obtain a fine feature map. The network makes innovations in the fusion of features at different levels, which makes the network recognition performance superior. The above methods have achieved certain results in the field of remote sensing CD. However, the accurate extraction of effective feature representations and the adequate fusion of feature information at different scales are still research challenges in the field of remote sensing CD. For the benefit of retrieval, a summary of the above-mentioned methods is presented in Table 1.

In this paper, we propose a Multi-Attention Guided Feature Fusion Network (MAFF-Net) for remote sensing images to address the above problems effectively. The main contributions of this article are as follows:

1. We propose the Feature Enhancement Module (FEM), which solves the problem that the features extracted from the backbone network have much interference information and the feature representation is not clear enough. The FEM captures not only cross-channel information but also direction-aware and location-sensitive information, which helps the model to locate the region of interest more accurately and enhance the representation of changing region features.
2. To solve the problem of inadequate feature fusion and insufficient feature communication in different layers or scales, we designed the attention-based Feature Fusion Module (FFM), which is divided into FFM_ S1 and FFM_S2 according to the input feature maps. FFM_S1 fuses the high-level feature maps with the low-level feature maps by a cross-layer approach. This cross-layer feature fusion approach is of great benefit to highlight the spatial consistency of objects. FFM_S2 fuses two feature maps of the same scale, and it should be noted that one is the feature map of T1 and one is the feature map of T2. The role of FFM_S2 is to fully fuse the feature maps of the bi-temporal image pairs to obtain a better change map.
3. We propose a Refinement Residual Block (RRB) using a residual structure, which can compensate for the shortcomings of using a single 3×3 convolutional kernel to refine the feature representation method.

We tested the model on three publicly available remote sensing image datasets. The experimental results validate the effectiveness of our proposed algorithm. The remainder of this article is organized as follows: Section 2 describes the proposed method in detail. In Section 3, corresponding experiments are designed to verify the effectiveness of the method in this article, and the experimental results are analyzed and discussed. Section 4 draws some conclusions about our method.

## 2. Methodology

In this section, a detailed description of the network proposed for the remote sensing image CD task is presented. First, the backbone of the architecture is described. Second, a detailed description of the proposed FEM is presented. Next, the attention-guided feature fusion mechanism is the focus of this section description, and these modules are described separately in this section. Then, the RRB proposed in this paper is introduced. Finally, the final prediction results are generated by applying convolutional operations [48,49] on the final fused feature maps.

### 2.1. Network Architecture

**Figure 1:**
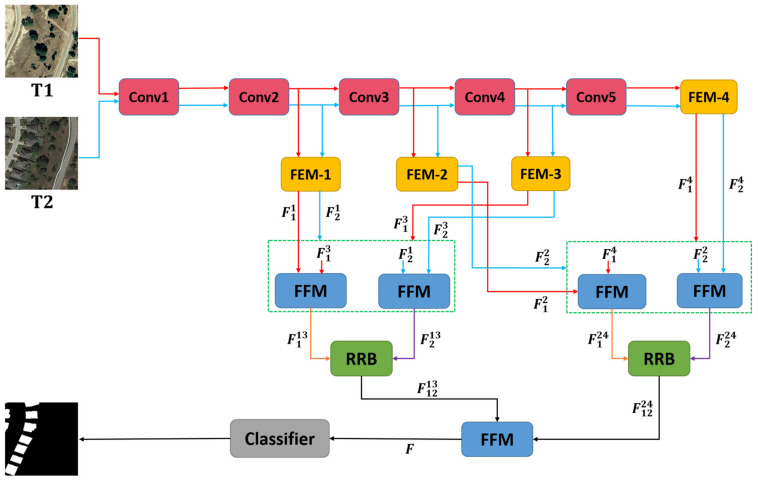
Architecture of the proposed MAFF-Net network. The green dotted box shows the cross-layer fusion strategy. $\left(F_{1}^{1},F_{1}^{2},F_{1}^{3},F_{1}^{4}\right)$ and $\left(F_{2}^{1},F_{2}^{2},F_{2}^{3},F_{2}^{4}\right)$ denote the two sets of features updated by the FEM.

The overall structure of the proposed network in this paper is shown in Figure 1. The proposed network uses ResNet18 as its backbone network. Based on some previous work [42,50,51], the proposed network modifies Res-Net18 by removing the last max-pooling layer and the fully connected layer and retaining the layers in the first five convolutional blocks (Conv1 to Conv5).

First, the bi-temporal image pairs $\left(T1,T2\right)$ are input to the feature extraction network to obtain sets of feature maps, $\left(F_{T1}{}_{1}^{0},F_{T1}{}_{1}^{1},F_{T1}{}_{1}^{2},F_{T1}{}_{1}^{3},F_{T1}{}_{1}^{4}\right)$ and $\left(F_{T2}{}_{2}^{0},F_{T2}{}_{2}^{1},F_{T2}{}_{2}^{2},F_{T2}{}_{2}^{3},F_{T2}{}_{2}^{4}\right)$. For each set of feature maps, the proposed method uses only the last four feature maps. These feature maps are then fed into the Feature Enhancement Module (FEM) according to their respective scales to obtain two sets of updated feature maps, $\left(F_{1}^{1},F_{1}^{2},F_{1}^{3},F_{1}^{4}\right)$ and $\left(F_{2}^{1},F_{2}^{2},F_{2}^{3},F_{2}^{4}\right)$. Next, the cross-layer feature fusion strategy is employed for each of the two updated feature maps. It should be noted here that our cross-layer feature fusion strategy targets different scale features of the same image. Specifically, take image *T*1 as an example. First, bilinear up-sampling [52,53,54] and convolution operations are performed on high-level features $F_{1}^{3}\in R^{4C\times H/4\times W/4}$ to obtain $F_{1}^{3}\in R^{C\times H\times W}$, where H×W is the size of the feature map $F_{1}^{1}\in R^{C\times H\times W}$ and C is the channel dimension of $F_{1}^{1}$. Then, the feature maps $F_{1}^{1}$ and $F_{1}^{3}$ of the *T*1 image are concatenated to obtain feature $F_{1}^{13}\in R^{2C\times H\times W}$. $F_{1}^{13}$ is input to the convolutional block attention module (CBAM) [55] and then output to $F_{1}^{13}\in R^{C\times H\times W}$ after using 3×3 convolution on it. The same method is used to fuse $F_{1}^{2}\in R^{2C\times H/2\times W/2}$ and $F_{1}^{4}\in R^{8C\times H/8\times W/8}$ of *T*1 to obtain $F_{1}^{24}\in R^{2C\times H/2\times W/2}$. With the FFM module, four feature maps $F_{1}^{13}$, $F_{2}^{13}$, $F_{1}^{24}$, and $F_{2}^{24}$ were obtained. Depending on the corresponding scales, the fused feature map pairs, $\left(F_{1}^{13},\;F_{2}^{13}\right)$ and $\left(F_{1}^{24},\;F_{2}^{24}\right)$, are fed into our proposed RRB to further refine the feature representation to obtain $F_{12}^{13}\in R^{C\times H\times W}$ and $F_{12}^{24}\in R^{2C\times H/2\times W/2}$, respectively. Finally, the two feature maps, $F_{12}^{13}$ and $F_{12}^{24}$, are sent to the FFM for final fusion. The prediction map is obtained after applying a pixel classifier (equipped with the sequence 3×3 Conv, batch normalization (BN) [56], and ReLU [57]).

### 2.2. Feature Enhancement Module

**Figure 2:**
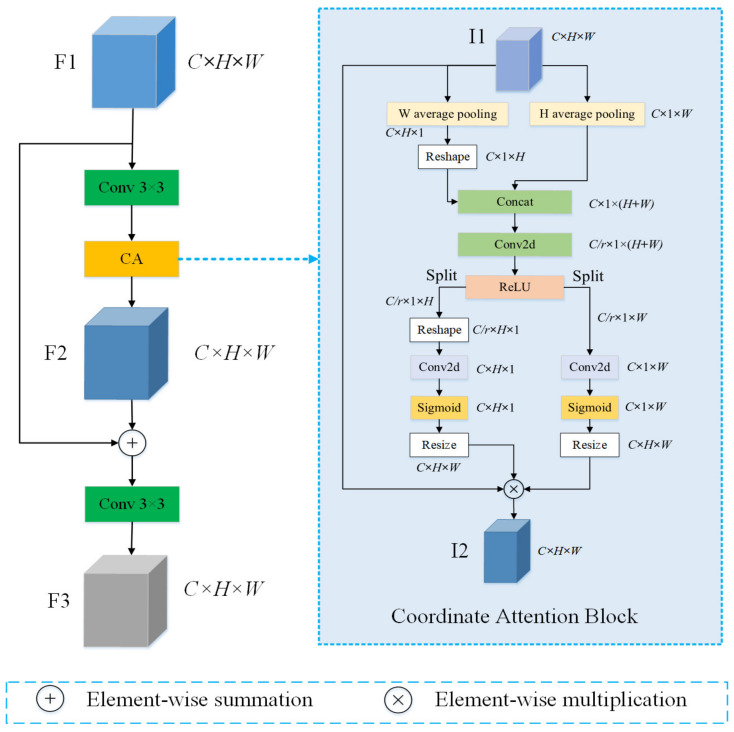
Feature Enhancement Module (FEM). “W average pooling” and “H average pooling” refer to 1D horizontal global average pooling and 1D vertical global average pooling, respectively. The r indicates the reduction ratio, where r is set to 16. The Reshape operation permutes the Dimension of the tensor. The Resize operation extends the tensor to the same size as the input I1.

The existing CD methods for HR remote sensing images have received less attention to the position information and channel relationships. HR remote sensing images have rich location-spatial information. To obtain accurate position information, a Feature Enhancement Module (FEM) based on coordinate attention (CA) is proposed in this paper to obtain the accurate location information and channel relationships of HR remote sensing images. The module can consider both position information and channel information. The structure of the FEM is shown in Figure 2.

In Figure 2, first, a 3×3 convolution operation is performed on the input *F*1. Then it is fed into the CA block to obtain the weighted feature map, $F2\in R^{C\times H\times W}$. Feature maps *F*1 and *F*2 are merged into one feature map by element-wise summation, and a 3×3 convolution operation is used to obtain *F*3.

In Figure 2, the coordinate attention module encodes *H* and *W* respectively. In the HR remote sensing image, for a given position $\left(i,j\right)$, its pixel value on channel *c* is $x_{c}\left(i,j\right)$. The *H* average pooling output of the *c*-th channel at height *h* is as Equation (1) [46]:

(1) $$z_{c}^{h}\left(h\right)=\frac{1}{W}\sum_{0\leq i<W}x_{c}\left(h,i\right)$$

Similarly, the *W* average pooling output of the *c*-th channel at width *w* is as Equation (2) [46]:

(2) $$z_{c}^{w}\left(w\right)=\frac{1}{H}\sum_{0\leq j<H}x_{c}\left(j,w\right)$$

Then, the Reshape operation is used to permute the dimensionality of the $z_{c}^{h}$ tensor to be the same as that of the $z_{c}^{w}$ tensor. Next, the coordinate attention module uses concatenation, convolution, and activation function operations. The related definition is as Equation (3) [46]:

(3) $$f=\delta \left(F_{C}\left(\left[z_{c}^{h},\;z_{c}^{w}\right]\right)\right)$$

where [,] indicates a concatenation operation, $F_{C}$ indicates a 1 × 1 convolution operation, and δ indicates the ReLU activation function. f is the output feature map of the ReLU layer.

After the split operation, f can be decomposed into $f^{h}\in R^{C/r\times 1\times H}$ and $f^{w}\in R^{C/r\times 1\times W}$. The reshape operation is used again to permute the dimension of the tensor $f^{h}$ to obtain $f^{h}\in R^{C/r\times H\times 1}$. Next, two 1×1 convolutional transforms $F_{h}$ and $F_{w}$ are used to transform $f^{h}$ and $f^{w}$ into tensor with the same number of channels as the input I1, respectively. Then, applying the sigmoid activation function [58] to the tensors updated by $F_{h}$ and $F_{w}$, respectively, two outputs are obtained as shown in Equation (4) and Equation (5) [46]:

(4) $$g^{h}=\sigma \left(F_{h}\left(f^{h}\right)\right)$$

(5) $$g^{w}=\sigma \left(F_{w}\left(f^{w}\right)\right)$$

where σ indicates sigmoid activation function. The Resize operation expands the size of $g^{h}\in R^{C\times H\times 1}$ and $g^{w}\in R^{C\times 1\times W}$ to the same size as the input $I1\in R^{C\times H\times W}$, respectively, and the $g^{h}$ and $g^{w}$, after being Resized, are used as attention weights. Finally, the output feature map I2 of the CA block is defined as Equation (6) [46]:

(6) $$y_{c}\left(i,j\right)=\left(x_{c}\left(i,j\right)\times g_{c}^{h}\left(i\right)\right)\times g_{c}^{w}\left(j\right)$$

where *c* is the *c*-th channel, $g_{c}^{h}\left(i\right)$ is the weight of the *i*-th position in the *H* direction, $g_{c}^{w}\left(j\right)$ is the weight of the *j*-th position in the *W* direction, and $y_{c}\left(i,j\right)$ is the value of the output feature map I2.

### 2.3. Feature Fusion Module

With the study of deep learning-based CD, it has been found that the CD task is unsatisfactory if it relies only on simple feature extraction networks. On the one hand, this is because simple feature extraction networks cannot eliminate semantic interference such as seasonal appearance differences and cannot accurately label change regions in the presence of diverse object shapes and complex boundaries. On the other hand, it is not fully exploited to multi-scale information, and the fusion of multi-scale features to make them communicate can help our network improve its performance.

**Figure 3:**
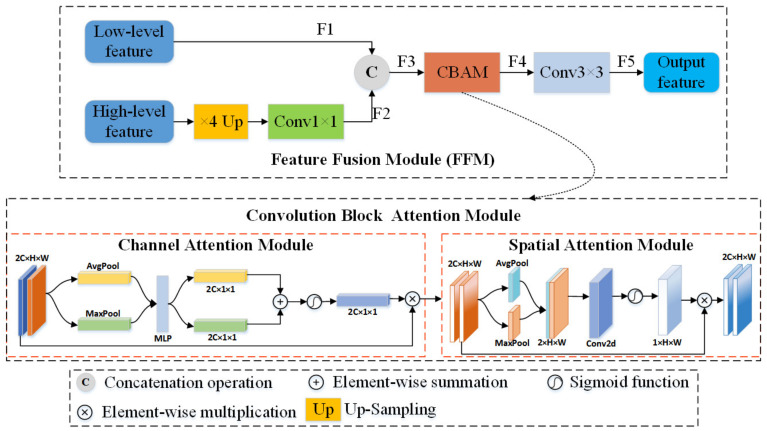
Feature Fusion Module (FFM). *F*1–*F*5 represent the feature maps that are output by different blocks.

Therefore, as shown in Figure 1, an attention-based Feature Fusion Module (FFM) is introduced into the CD network. The detail of the FFM is shown in Figure 3.

The proposed FFM is slightly different at different stages. The FFM whose input features are from FEM is named FFM_S1, and the FFM whose input features are from RRB is named FFM_S2. Specifically, the difference between FFM_S1 and FFM_S2 lies in the input part. The inputs of FFM_S1 are two feature maps of different scales of one image, while the input of FFM_S2 is two feature maps of the same scale of two images.

After FEM processing, two sets of updated feature maps, $\left(F_{1}^{1},F_{1}^{2},F_{1}^{3},F_{1}^{4}\right)\in T1$ and $\left(F_{2}^{1},F_{2}^{2},F_{2}^{3},F_{2}^{4}\right)\in T2$, were obtained. For FFM_S1, the inputs are the feature map pairs $\left(F_{1}^{1},F_{1}^{3}\right)$ and $\left(F_{1}^{2},F_{1}^{4}\right)$ and $\left(F_{2}^{1},F_{2}^{3}\right)$ and $\left(F_{2}^{2},F_{2}^{4}\right)$, respectively. Figure 3 shows FFM_S1, and the structure of FFM_S2 is not drawn separately because the two only have different inputs. However, it should be emphasized here that FFM_S2, which has two input feature maps of the same scale, does not distinguish between high-level features and low-level features, and also does not need to up-sample high-level features such as FFM_S1.

The next step is to describe FFM_S1. After experiments, it is found that the fusion of features by cross-layer is more effective. This may be because the high-level features will lose some semantic information carried by the original image or low-level features, such as some edge features, as the number of convolution layers increases, and the fusion with low-level features can compensate for this deficiency. At the same time, the semantic information carried by the feature maps between neighboring layers is not so obviously different, so the fusion method by cross-layer plays a role. For an original image *T*1, feature map pairs $\left(F_{1}^{1},F_{1}^{3}\right)$ and $\left(F_{1}^{2},F_{1}^{4}\right)$ are fed into FFM_S1, respectively. For original image *T*2, feature map pairs $\left(F_{2}^{1},F_{2}^{3}\right)$ and $\left(F_{2}^{2},F_{2}^{4}\right)$ are fed into FFM_S1, respectively. As shown in Figure 3, the high-level feature needs an up-sampling operation to make the feature map shape consistent with the low-level feature. Next, one 1×1 convolution is used to obtain the feature map $F2\in R^{C\times H\times W}$. The two inputs $F1\in R^{C\times H\times W}$ and F2 are concatenated to obtain the feature map $F3\in R^{2C\times H\times W}$. The resulting feature map can be viewed as a feature map with different channels. The calculation process of F3 is shown in Equation (7):

(7) $$F3=\left[Conv\left(Up\left(F1\right)\right),F2\right]$$

where *Conv* denotes the 1×1 convolution, and [.,.] denotes the concatenation operation. Considering that this direct aggregation of features in cross-layer does not yet communicate well in the channel and spatial dimensions, feed F3 to the *CBAM*. *CBAM* is an attention module consisting of the channel and spatial attention. It considers both the importance of pixels in different channels and the importance of pixels in different positions in the same channel. The *CBAM* outputs the feature map $F4\in R^{2C\times H\times W}$. Then, the 3×3 convolution block is used, the main purpose of which is to recover the channels of the aggregated feature map to the number of channels of the input feature map. The above calculation process is shown in Equation (8):

(8) $$F4=Conv\left(CBAM\left(F3\right)\right)$$

where *Conv* denotes the 3×3 convolution block. Next, in two subsubsections, two parts of *CBAM*, namely the channel attention module and the spatial attention module, are described in detail.

#### 2.3.1. Channel Attention Module

In the Channel Attention Module (CAM), the vectors described as A$F_{avg}^{ca}\in R^{B\times C\times 1}$ and $F_{max}^{ca}\in R^{B\times C\times 1}$ are obtained by the average-pooling and max-pooling operations, respectively. Then, each of them is input to the shared multi-layer perceptron (MLP) with one hidden layer, respectively, to get two vectors, and the two vectors are merged to one feature vector by element-wise summation. After sigmoid activation, the feature map of the CAM is finally obtained. This is shown in Equation (9) [55]:

(9) $$M_{ca}\left(D\right)=\delta \left(FC_{1}\left(FC_{0}\left(F_{avg}^{ca}\right)\right)+FC_{1}\left(FC_{0}\left(F_{max}^{ca}\right)\right)\right)$$

where $FC_{0}$ and $FC_{1}$ denote the convolution operation in MLP and δ denotes the sigmoid function. The CAM compresses the feature map spatial dimensions to obtain a one-dimensional vector before manipulating it. Channel attention is concerned with what is significant on this feature map. The average-pooling has feedback for every pixel point on the feature map, while max-pooling has feedback for gradients only where the response is greatest in the feature map when performing gradient backpropagation calculations.

#### 2.3.2. Spatial Attention Module

In the Spatial Attention Module (SAM), it is the feature map output from the CAM that is used as input. First, do a max-pooling and average-pooling based on the channel to get the element-wise summation, and then a concatenation operation is performed on the two layers. Then, convolution is performed and reduced to 1 channel, and then the feature map output from the SAM is obtained by sigmoid activation. This is given by Equation (10) [55]:

(10) $$M_{sa}\left(D^{ca}\right)=\delta \left(f^{7\times 7}\left(Cat\left(F_{avg}^{sa},F_{max}^{sa}\right)\right)\right)$$

where *Cat* is the concatenation operation, $f^{7\times 7}$ represents a convolutional layer with a filter size of 7×7, and δ denotes the sigmoid function. The SAM is a channel compression mechanism that performs average-pooling and max-pooling in the channel dimension respectively. The max-pooling operation is to extract the maximum value on the channel, and the number of extractions is H×W. The average-pooling operation is to extract the average value on the channel, and the number of extractions is also H×W. Thus, a *2*-channel feature map can be obtained.

### 2.4. Refinement Residual Block

**Figure 4:**
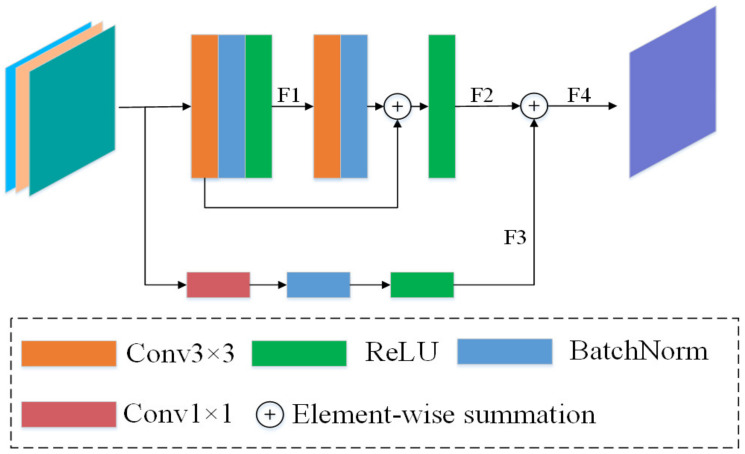
Refinement Residual Block (RRB). *F*1–*F*4 represent the feature maps that are output by different blocks.

The use of a single 3×3 convolutional kernel has some shortcomings in refining the feature representation. Inspired by Yu et al. [59], a Refinement Residual Block (RRB) is introduced to modify the channels of the aggregated feature map to be consistent with the input feature map and further refine the feature representation before the final feature fusion using FFM_S2. Its structure is shown in Figure 4.

As can be seen in Figure 4, the RRB has three inputs, one of which is the difference map of two feature maps. The three feature maps are first subjected to a concatenation operation, followed by two consecutive convolution blocks, each consisting of *Conv* 3×3, BN, and ReLU. The two convolution blocks output the feature maps $F1\in R^{C\times H\times W}$ and $F2\in R^{C\times H\times W}$, respectively. Here, it should be noted that the number of channels of each convolutional block output is different. In addition, the module adds additional residual connections with the 1×1 convolutional layers for obtaining some additional spatial information of the remote sensing images. Finally, the four feature maps are subjected to element-wise summation and the final output feature map $F4\in R^{C\times H\times W}$ is obtained.

### 2.5. Loss Function

In the training stage, a cross-entropy loss function optimized by Chen et al. [43] is used, which minimizes the cross-entropy loss to optimize the network parameters. Formally, the loss function is defined as Equation (11) [43]:

(11) $$L=\frac{1}{H_{0\times }W_{0}}\sum_{h=1,w=1}^{H,W}l\left(P_{hw},Y_{hw}\right)$$

where $l\left(P_{hw},y\right)=-log\left(P_{hwy}\right)$ is the cross-entropy loss and $Y_{hw}$ is the label for the pixel at location $\left(h,w\right)$ [43].

## 3. Experiments and Results

In this section, the proposed network MAFF-Net is evaluated on three publicly available benchmark datasets to demonstrate its effectiveness. First, the details of the three datasets, the CDD dataset [60], the LEVIR-CD dataset [42], and the WHU-CD dataset [61], are introduced. Next, the implementation details are presented, including the experimental environment and evaluation metrics. Then, seven state-of-the-art (SOTA) comparison methods are introduced. In this section, quantitative and qualitative analyses of these methods are presented on three datasets.

### 3.1. Datasets and Settings

**Figure 5:**
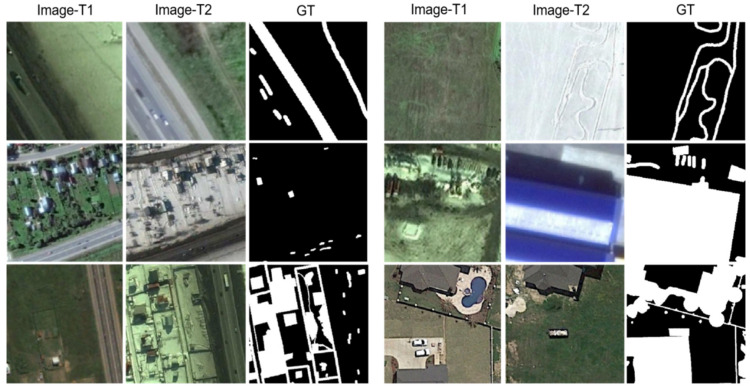
Illustration of samples from CDD. (Image-T1) and (Image-T2) indicate the bi-temporal image pairs. (GT) indicates the ground truth.

The CDD dataset has three types of images, synthetic images with no relative movement of objects, synthetic images with less relative movement of objects, and real remote sensing images with seasonal changes (obtained from Google Earth). In this paper, a subset of remote sensing image data with seasonal changes is selected. This subset has 16,000 images with an image size of 256×256 pixels, of which 10,000 images are used as the training set, 3000 images as the validation set, and 3000 images as the test set. As shown in Figure 5, the change scenarios of this dataset include building changes, road changes, and vehicle changes. The data set was considered for different sizes of objects.

**Figure 6:**
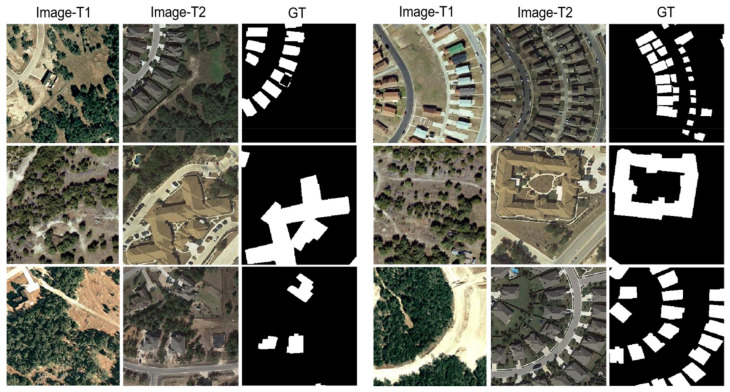
Illustration of samples from LEVIR-CD. (Image-T1) and (Image-T2) indicate the bi-temporal image pairs. (GT) indicates the ground truth.

LEVIR-CD contains 637 very high resolution (VHR, 0.5 m/pixel) Google Earth image patch pairs, 1024×1024 pixels in size. These bitmap images spanning 5 to 14 years have significant land-use changes, especially building growth. LEVIR-CD covers various types of buildings such as villas, high-rise apartments, small garages, and large warehouses. The fully annotated LEVIR-CD contains a total of 31,333 individual instances of change construction. As shown in Figure 6, each sample is cropped into 16 small patches of size 256×256, generating 7120 image patch pairs for training, 1024 for validation, and 2048 for testing.

**Figure 7:**
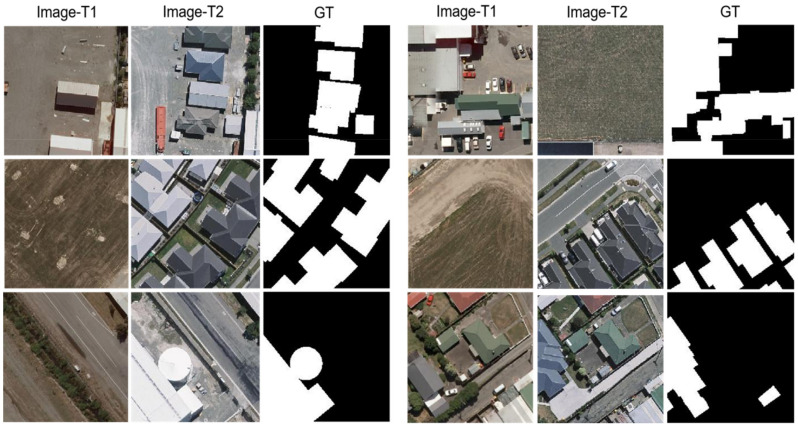
Illustration of samples from WHU-CD. (Image-T1) and (Image-T2) indicate the bi-temporal image pairs. (GT) indicates the ground truth.

The third dataset is named the WHU-CD dataset, which is a CD dataset of public buildings. The dataset covers the area where the 6.3 magnitude earthquake occurred in February 2011 and has been reconstructed in the following years. It consists of a pair of HR (0.075 m) aerial images of size 32,507×15,354. Considering that the authors of the original paper did not provide a solution for data segmentation, as shown in Figure 7, the solution of cropping the image into small pieces of size 224×224 was finally chosen, and dividing them into three random parts: 7918/987/955 for training/validation/testing, respectively.

### 3.2. Evaluation Metrics and Settings

For quantitative assessment, three indices, namely the *F*1-score (*F*1), *Kappa* coefficient (*Kappa*), and overall accuracy (*OA*) are used as the evaluation metrics. These three indices can be calculated as follows:

(12) $$P=\frac{\mathrm{TP}}{\mathrm{TP}+\mathrm{FP}}$$

(13) $$R=\frac{\mathrm{TP}}{\mathrm{TP}+\mathrm{FN}}$$

(14) $$F1=\frac{2}{P^{-1}{+R}^{-1}}$$

(15) $$\mathrm{OA}=\frac{\mathrm{TP}+\mathrm{TN}}{\mathrm{TP}+\mathrm{FP}+\mathrm{TN}+\mathrm{FN}}$$

(16) $$PRE=\frac{\left(TP+FN\right)\times \left(TP+FP\right)+\left(TN+FP\right)\times \left(TN+FN\right)}{{\left(\mathrm{TP}+\mathrm{TN}+\mathrm{FP}+\mathrm{FN}\right)}^{2}}$$

(17) $$Kappa=\frac{\mathrm{OA}\;-\;\mathrm{PRE}}{1\;-\;\mathrm{PRE}}$$

where *OA* and *PRE* denote the overall accuracy and expected accuracy, respectively. The *TP*, *FP*, *TN*, and *FN* are the number of true positives, false positives, true negatives, and false negatives, respectively. 

We implemented our proposed method with PyTorch, supported by NVIDIA CUDA with a GeForce GTX 2080Ti GPU. In the training stage, the feature extraction backbone of the proposed MAFF-Net is initialized from ResNet18. We used the Adam ($\beta_{1}=0.5$, $\beta_{2}=0.9$) optimizer and the entire training period was set to 200 epochs. The initial learning rate is 0.001 in the first 100 epochs, in the next 100 epochs, the value of the learning rate decays linearly to 0. Considering the GPU size, we set the batch size to 8 to facilitate GPU training.

### 3.3. Comparison of Experimental Results

In this section, the performance of the different methods is compared on the three datasets CDD, LEVIR-CD, and WHU-CD, respectively. The advantages and disadvantages of each method are further described based on the results of the quantitative and qualitative analyses. In addition, an ablation study is performed on the proposed method to compare and analyze the effectiveness of each of its modules.

#### 3.3.1. Comparison Methods

To verify the effectiveness and superiority of our methods, we selected seven methods that are represented in the CD task and compared the performance of these methods in CDD, LEVIR-CD, and WHU-CD, respectively, and a brief description of the selected methods is as follows:

1. CD-Net [62] combines the multi-sensor fusion SLAM and fast density 3D reconstruction for coarse alignment of image pairs followed by deep learning methods for pixel-level CD.
2. FC-EF [38] refers to early fusion with full convolution. It concatenates the two input images before feeding them into the network, treating them as different channels of one image. It is then fed into a standard U-Net.
3. FC-Siam-conc [38] connects three feature maps from the two encoder branches and the corresponding layer of the decoder.
4. FC-Siam-diff [38] first finds the absolute value of the difference between the feature maps of the two decoder branches and then makes a skip-connection to the corresponding layer of the decoder.
5. DASNet [44] is a CD model based on a dual-attentive fully convolutional twin neural network and proposes a weighted double-margin contrastive loss (WDMC) to be able to solve the sample imbalance problem.
6. IFN [45] first uses the two Siamese network architectures as the raw images feature extraction network. To enhance the integrity of change map boundaries and internal densities, multi-level depth features are fused with image difference map features by an attention mechanism.
7. STANet [42] proposes a new spatial-temporal attention neural network based on twin networks. The network exploits spatial-temporal dependence and designs a CD self-attentive mechanism to model spatial-temporal relations. A new HR remote sensing image dataset, LEVIR-CD, is also proposed.

#### 3.3.2. CDD Dataset

**Figure 8:**
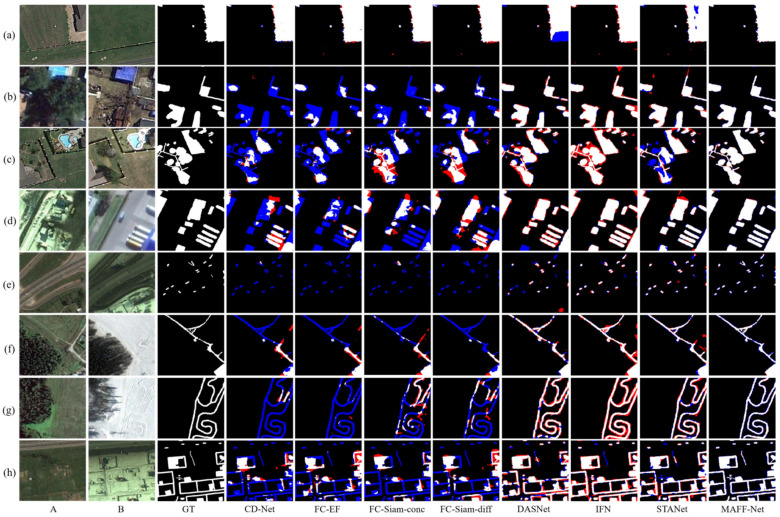
Illustration of a qualitative comparison on dataset CDD. (**a**–**h**) indicate samples from CDD and the change maps obtained with different methods. The white color indicates the changes that were correctly detected. Black indicates that no changes have been correctly detected. Red indicates false alarms. Blue indicates unpredicted changes.

**Figure 9:**
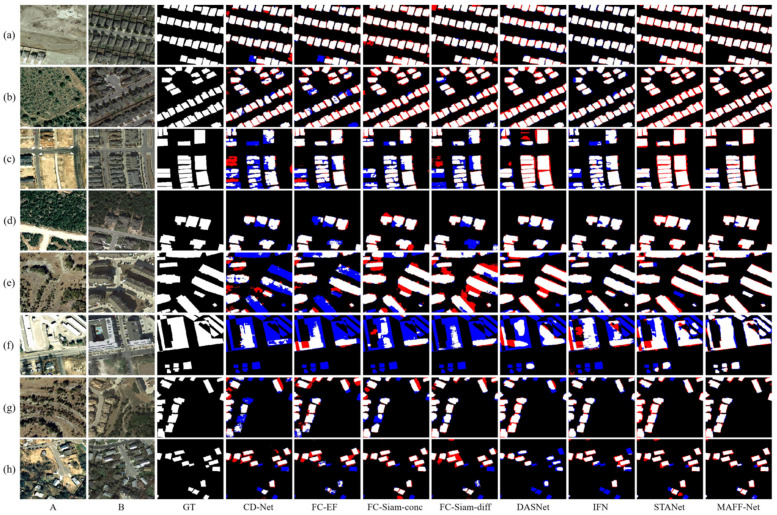
Illustration of a qualitative comparison on dataset LEVIR-CD. (**a**–**h**) indicate samples from LEV-IR-CD and the change maps obtained with different methods. The white color indicates the changes that were correctly detected. Black indicates that no changes have been correctly detected. Red indicates false alarms. Blue indicates unpredicted changes.

**Figure 10:**
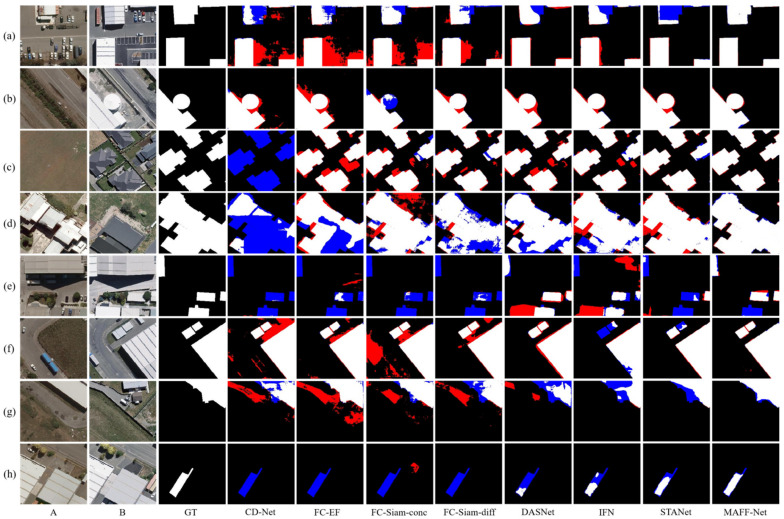
Illustration of a qualitative comparison on dataset WHU-CD. (**a**–**h**) indicate samples from WHU-CD and the change maps obtained with different methods. The white color indicates the changes that were correctly detected. Black indicates that no changes have been correctly detected. Red indicates false alarms. Blue indicates unpredicted changes.

For quantitative comparison, we calculated and summarized the evaluation metrics for CDD, LEVIR-CD, and WHU-CD, as shown in Table 1, Table 2 and Table 3, respectively. To compare the performance of each method more visually, we visualized the test results of each method on the three data sets, as shown in Figure 8, Figure 9 and Figure 10, respectively. The white color indicates the changes that were correctly detected. Black indicates that no changes have been correctly detected. Red indicates false alarms. Blue indicates unpredicted changes.

As can be seen from Table 2, the proposed MAFF-Net reached the first on *F*1, *Kappa*, and *OA* on the CDD dataset. This also indicates that the proposed network performs optimally on this dataset. It is also evident from Figure 8 that the proposed network can better mark the change region, while there are few cases of wrong and missing detections. Specifically, as can be seen from the data in Table 2, CD-Net, which does not pay attention to the connections and interactions between multi-scale features, performs relatively poorly in the three evaluation metrics, 14.6% lower than the proposed MAFF-Net in terms of *F*1 score. This is somewhat related to its fewer network levels and relatively simple structure. Considering early fusion and late fusion strategies separately and using skip-connected encoding-decoding, the baselines of FC-EF, FC-Siam-conc, and FC-Siam-diff achieve better performance with their compact and efficient structures. Among these three baselines, the late fusion baseline shows a clear advantage over the early fusion baseline. The fusion of feature maps using bi-temporal image pairs with their difference maps achieves better results than the fusion of feature maps using only bi-temporal image pairs. FC-Siam-Diff scores 0.8%, 0.9%, and 0.1% higher than FC-Siam-conc on *F*1, *Kappa*, and *OA*, respectively. This is because the original image coding features are preserved as much as possible while obtaining the difference maps. This helps the network to achieve better performance.

Based on the attention mechanism, which can further focus on the information exchange between feature maps, DASNet works better than FC-EF. IFN pays more attention to the connection and interaction of multi-scale information. It introduces channel attention and spatial attention and uses a post-fusion strategy for deep supervision. Its *F*1 and *Kappa* scores reached 90.1% and 89.2%, respectively. STANet proposes a spatial-temporal attention module based on a feature pyramid to better adapt the network to the detection task of complex scenes, ranking second in all evaluation metrics. The proposed MAFF-Net achieves the highest level in all metrics, respectively. It is able to detect and label the change regions better than other methods because the network employs an attention-based cross-layer feature fusion strategy and also designs a refinement residual block to further improve the network detection performance.

Also, the qualitative analysis in Figure 8 allows for further analysis of the performance of each network. For visual analysis, eight challenging sets of bi-temporal images were selected and visualized. Each set of images contains different ranges of change regions or change scenes. Among the three FCN-based baselines, FC-Siam-conc and FC-Siam-diff can give better results than FC-EF. As can be seen in Figure 8, only a small number of change regions (Figure 8a) can be marked by FC-EF, but it performs poorly for smaller change regions and more complex scenes (Figure 8b–h). This is because it does not preserve the features of each original image, especially the shallow features, which makes the detected change regions significantly inaccurate. In general, the other two baselines perform better than FC-EF, as evidenced by the completeness of the information in the regions of change detected in the illustrations. However, they still suffer from many missed and false detections, such as Figure 8b–g. In particular, in Figure 8e, they do not detect the change region at all. Therefore, there is still potential for improvement. By introducing dual attention in the decoding stage, DASNet can detect most of the change regions. However, its detection performance for small change regions needs to be improved. For example, in Figure 8e, there are many missed regions in its detection results, and there are also false detection regions. This demonstrates that it is not yet quite accurate in terms of the boundaries and details of the change regions. In addition, it also does not perform well in Figure 8b,f,h with false detections and missed detections.

IFN and STANet are relatively more complete in terms of local detail because of the introduction of channels and spatial attention. However, they still have false positives and false negatives in detecting some very small target regions or edges, as shown in the red and blue regions in Figure 8c,e,g,h. The processing of some regions is too smoothed, and some edge information is ignored to some extent. The proposed MAFF-Net can better label the change regions and accurately detect the edges of the change regions. It can be seen from the exhibited samples that there are very few red and blue regions representing false and missed detections. In particular, the detection performance is well for small and complex change regions, as shown in Figure 8e–h, for example. This also demonstrates that the proposed network can detect the change regions accurately in general.

#### 3.3.3. LEVIR-CD Dataset

As can be seen from Table 3, the difference in performance between the three baselines of FCN is not significant, where the higher score among the three indicators is FC-Siam-Diff, with *F*1 and *Kappa* scores of 83.7% and 82.8%, respectively. DASNet, by introducing dual attention, improved the *F*1-score by about 0.9% compared to the three baselines of FCN. The *F*1 score of IFN reached third place with 86.2%, while the scores of *Kappa* and *OA* also performed well. However, the scores of all metrics are lower than those of STANet, which may be because STANet pays more attention to multi-scale information while introducing attention. By introducing an attention mechanism involving multiple scales, the proposed MAFF-Net improves the *F*1 score to 89.7%, which is better than other comparative methods. Moreover, *Kappa* and *OA* reached the highest values among the compared methods with 89.1% and 98.7%, respectively.

Figure 9 also illustrates the change maps on eight selected sets of bi-temporal images. The change regions in these images cover multiple scenes, areas, shapes, and distribution ranges. For multiple regularly shaped building changes in Figure 9a,b, the overall contours of the buildings are correctly detected. However, the detection results of the CD-Net and FC-EF methods still have obvious false detection and missed detection areas. Although STANet can locate the change region, the detection of more complex and small change regions is not entirely correct. For example, as shown in Figure 9f,h, the proposed MAFF-Net is more accurate than the other methods, as seen from the fewer regions marked in red and blue. For Figure 9a,b,d, the attention-based methods DASNet and STANet and the proposed attention-based guided cross-layer feature fusion network MAFF-Net are visually closer to the GT. For the more densely distributed change regions in Figure 9c, DASNet, STANet, and MAFF-Net maintain visual correctness, while MAFF-Net has fewer errors and can accurately detect and distinguish multiple dense change regions. However, for Figure 9f–h with more complex edges and smaller change regions, IFN, DASNet, and STANet do not perform well. On the contrary, MAFF-Net shows better adaptability, and it can accurately detect changing regions with complex shapes and small objects.

#### 3.3.4. WHU-CD Dataset

According to the data in Table 4, the performance of the methods with FCN as the baseline does not differ much. The double attention-based DASNet performs slightly better than IFN and STANet, with scores of 90.7%, 90.1%, and 99.0% for *F*1, *Kappa*, and *OA*, respectively. We attribute this to the fact that the weighted double-margin contrastive loss (WDMC) used by DASNet can solve the problem of sample imbalance. The proposed MAFF-Net achieved the best scores in all evaluation metrics compared to the other comparison methods. Compared with the method using FCN as the baseline, the proposed method obtained a 9.1%, 9.6%, and 1.0% increase in *F*1, *Kappa*, and *OA*, respectively. This also demonstrates the effectiveness of the proposed multi-attention-guided feature fusion-based method. Compared to DASNet, IFN, and STANet, the proposed method improves the gains for *F*1, *Kappa*, and *OA* by 1.7%, 2.0%, and 0.4%, respectively. Such gains are generated thanks to our fusion strategy that fully considers multi-scale features, while effectively exploiting the advantage of the attention to greatly improve the network performance.

For visual comparison, Figure 10 shows some typical CD results for the test samples in the WHU-CD dataset. As shown in Figure 10a,c–e,h, there are many missed detections and false detections in the compared methods. As shown in Figure 10c,e,h, CD-Net not only has false detections but also has many missed detection regions. The performance of the FCN-based FC-EF, FC-Siam-conc, and FC-Siam-diff have been improved and the missed detection regions are significantly reduced. However, they still have the same problems as CD-Net as shown in Figure 10d,e,h. In Figure 10e,h, the attention-based DASNet, IFN, and STANet do not perform well, with significant missed detection regions and some false detection regions. In terms of consistency with the GT, the proposed MAFF-Net achieves the best visual performance. Specifically, as shown in the samples in Figure 10, MAFF-Net significantly reduces the missed detections and has a very low false detection rate compared with other methods. In addition, the change maps generated by MAFF-Net have clearer and more accurate boundaries compared with other methods.

### 3.4. Ablation Study

**Figure 11:**
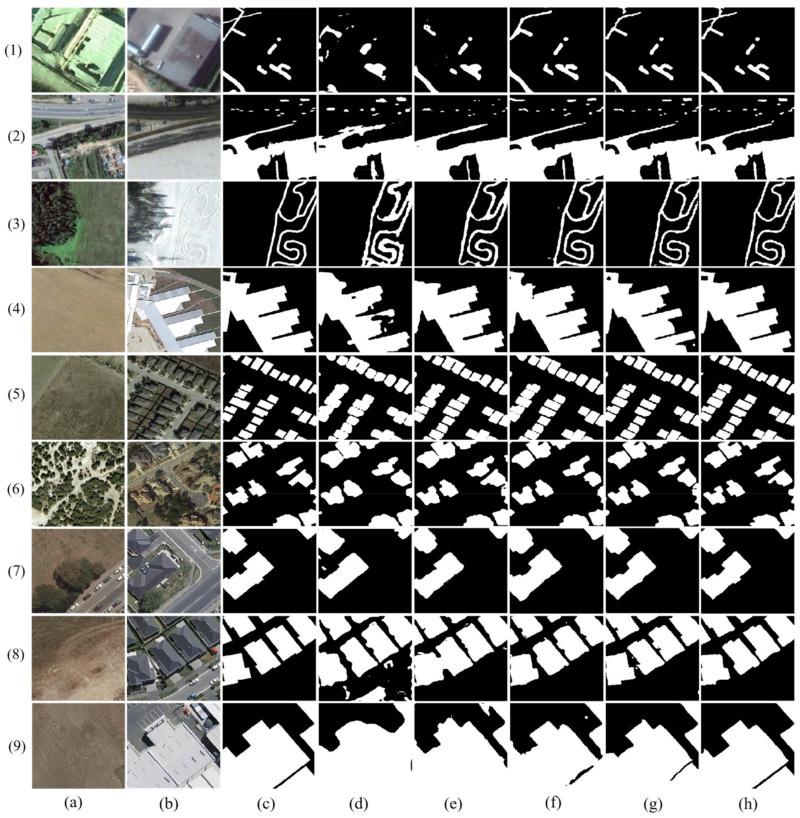
Visualization comparison plots of each network on different datasets in the ablation experiment. (**1**–**3**) indicate samples from the CDD dataset, (**4**–**6**) indicate samples from the LEVIR-CD dataset, and (**7**–**9**) indicate samples from the WHU-CD dataset. (**a**) Image T1. (**b**) Image T2. (**c**) Ground truth. (**d**) Baseline. (**e**) Baseline+FEM. (**f**) Baseline+FEM+FFM_S1. (**g**) Baseline+FEM+FFM_S1+RRB. (**h**) MAFF-Net (Baseline+FEM+FFM_S1+RRB+FFM_S2).

In the CD task, our proposed model achieves superior performance. To validate the effectiveness and feasibility of our proposed method, we conducted a series of ablation experiments on three datasets, CDD, LEVIR-CD, and WHU-CD, to verify that our model has advanced performance. We conducted five ablation experiments on three HR datasets, and in our experiments, the Baseline represents the ResNet18 network structure. In total, five ablation experiments were conducted in this paper: Baseline, Baseline+FEM, Baseline+FEM+FFM_S1, Baseline+FEM+FFM_S1+RRB, and the MAFF-Net (Baseline+FEM+FFM_S1+RRB+FFM_S2). As shown in Figure 11, the Baseline does not achieve good performance in detecting change regions, especially when the change region scene is more complex or the change region area is small (Figure 11d). Compared with the Baseline, the Baseline + FEM method obtains richer features after adding the FEM, which can help the network detect most of the change regions. It can be seen that the Baseline+FEM+FFM_S1 can effectively remove some irrelevant information (Figure 11f), while further capturing the change features and refining the feature representation. The FFM_S1 module adopts a cross-layer fusion strategy, which helps the model to fully fuse the features of high and low layers to achieve better feature representation. Compared with the Baseline+FEM method, the Baseline+FEM+FFM_S1 method detects more accurate and complete change regions. However, it can also be found that the method is slightly lacking when faced with small change regions or poorly characterized features (Figure 11f-1). Therefore, the Baseline+FEM+FFM_S1+RRB method aims to further refine the feature representation, which helps to detect smaller change features and improve the network performance. As can be seen by Figure 11g, the change map obtained by this method is already very close to the change region of the GT. Finally, the method proposed in this paper performs feature fusion feature maps to obtain a prediction map that is closest to the real change regions. As can be seen from Figure 11h, the change map obtained by the proposed method is very close to the GT, which also surfaces the effectiveness of the proposed method. Meanwhile, the proposed method shows good accuracy on three different datasets. By comparing the visualization results of each module, the effectiveness and accuracy of the MAFF-Net method proposed in this paper are effectively demonstrated.

In addition, we also performed statistics and comparisons on the *F*1, *Kappa*, and *OA* values of different methods. As shown in Table 5, the model achieves optimal performance when all innovation modules are added, which also proves the effectiveness of our proposed innovation modules.

In the Baseline+FEM method, as can be seen, there is a significant improvement in three indicators compared with the Baseline method. In the CDD dataset, *Kappa*, *F*1, and *OA* increased by 6.4%, 5.6%, and 1.5% compared with the Baseline, respectively. In the LEVIR-CD dataset, *Kappa*, *F*1, and *OA* were increased by 3.9%, 3.7%, and 0.4%, respectively, compared with the Baseline. In the WHU-CD dataset, *Kappa*, *F*1, and *OA* were increased by 4%, 3.9%, and 0.2%, respectively, compared with the Baseline. 

In the Baseline+FEM+FFM_S1 method, it can be seen that all metrics are improved compared to the baseline+FEM method. In the CDD dataset, *Kappa*, *F*1, and *OA* improve by 1.1%, 1%, and 0.3%, respectively, compared to the Baseline. In the LEVIR-CD dataset, *Kappa*, *F*1, and *OA* improved by 1.3%, 1.2%, and 0.1%, respectively, compared to the Baseline. In the WHU-CD dataset, *Kappa*, *F*1, and *OA* improved by 1.6%, 1.5%, and 0.1%, respectively, compared to the Baseline. We can see the improvement of all metrics on all datasets, indicating the innovation and validity of our proposed FFM_S1, while the joint use of FFM_S1 and FEM achieves better performance and makes the model more accurate.

In the Baseline+FEM+FFM_S1+RRB method, it can be seen that there are improvements in all metrics compared with the Baseline+FEM+FFM_S1 method. In the CDD dataset, *Kappa*, *F*1, and *OA* improve by 1.6%, 1.3%, and 0.3%, respectively, compared to the Baseline. In the LEVIR-CD dataset, *Kappa*, *F*1, and *OA* improved by 0.6%, 0.6%, and 0.1%, respectively, compared to the Baseline. In the WHU-CD dataset, *Kappa*, *F*1, and *OA* improved by 0.6%, 0.5%, and 0.1%, respectively, compared to the Baseline+FEM+FFM_S1. We can see the improvement in all metrics on all datasets, indicating that our proposed RRB enhances the feature representation of the feature map, while the combined use of FFM_S1, FEM, and RRB leads to better performance of the model.

In the Baseline+FEM+FFM_S1+RRB+FFM_S2 method, it can be seen that all metrics are improved compared to the baseline+FEM+FFM_S1+RRB approach. In the CDD dataset, *Kappa*, *F*1, and *OA* improved by 0.6%, 0.6%, and 0.2%, respectively, compared to the Baseline. In the LEVIR-CD dataset, *Kappa* and *F*1 improved by 0.9% and 0.9%, respectively, compared to the Baseline. In the WHU-CD dataset, *Kappa*, *F*1, and *OA* improved by 0.6%, 0.5%, and 0.1%, respectively, compared to the Baseline+FEM+ FFM_S1+RRB. We can see the improvement of all the metrics on all datasets, indicating our proposal that FFM_S2 has a facilitating effect in fusing multi-scale feature information exchanges, while FFM_S1 and FFM_S2 have a mutual facilitating effect in feature extraction, and also, it is known experimentally that MAFF-Net helps the network fuse multi-scale features to achieve multi-scale information communication, which can improve the performance of the network.

### 3.5. Efficiency Analysis of the Proposed Network

**Figure 12:**
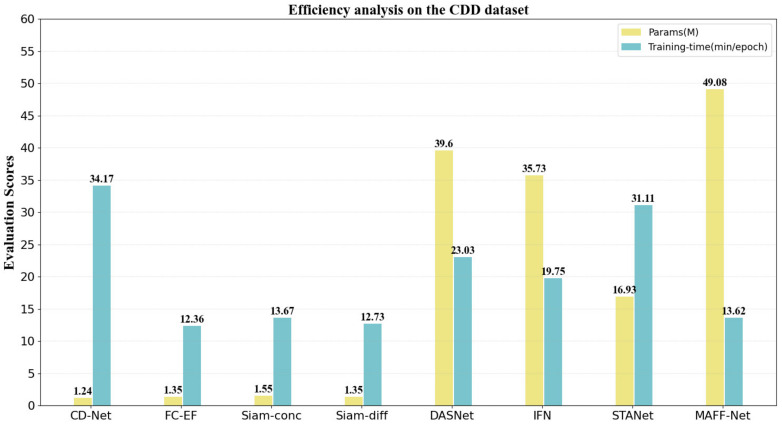
Illustration of an efficiency analysis of the comparison methods.

Although the proposed network MAFF-Net achieves encouraging performance, it has some potential limitations. The computational complexity of MAFF-Net is relatively high and the number of parameters is large. This is not friendly to devices and applications with limited resources. In this section, the parameter amount (take M as the unit) and the training time of an epoch (take min/epoch as the unit) are used as quantitative indicators for evaluation. As shown in Figure 12, the number of trainable parameters of MAFF-Net is 49.08 million, which is the largest among the compared methods. However, from another perspective, the training efficiency of the proposed MAFF-Net is also relatively impressive. Compared with STANet and DASNet, the training time of the proposed method is reduced by 56.22% and 40.86%, respectively, which makes the proposed method more valuable in practical applications under the same equipment conditions.

Though the number of training parameters and training time is comprehensive, the proposed method has space for improvement and enhancement in the future. For example, model compression can be performed in the proposed network, employing pruning and knowledge distillation [63,64] to reduce the size of the model.

## 4. Conclusions

In this paper, we propose a novel feature fusion network for remote sensing image CD tasks. To enhance the feature representation, we propose a Feature Enhancement Module (FEM), which introduces coordination attention (CA) that can capture long-range dependencies with precise location information while modeling inter-channel relationships. The FEM helps the network to further refine the features extracted by the backbone network ResNet18. The quantitative and qualitative analysis of the ablation study shows that the performance of the FEM on the Baseline is improved, which demonstrates the reasonability and effectiveness of the FEM. Considering that layer-by-layer feature fusion may lose part of the semantic information, we propose an FFM employing a cross-layer feature fusion strategy. The FFM uses semantic cues in the high-level feature map to guide feature selection in the low-level feature map. In addition, to highlight changing regions and suppress useless features, we introduce a CBAM in the FFM, which combines the advantages of channel attention and spatial attention, allowing the model to learn which region to focus on and pay more attention to critical information. Depending on the input features, we classified FFM into FFM_S1 and FFM_S2, both of which further enhance the feature fusion effect. Based on the ablation study in Section 3, we can see that the FFM significantly improves the performance of the network. To compensate for the shortcomings of using a single convolutional kernel for feature refinement, we propose a Refinement Residual Block (RRB) that employs a residual structure. The RRB changes the number of channels of the aggregated features and uses convolutional blocks to further refine the feature representation. Based on the comparison results between the proposed MAFF-Net and other methods in quantitative and qualitative analysis, the proposed method is able to efficiently detect changing regions and has a strong ability to select features through a feature fusion strategy guided by multiple attention mechanisms. On the three publicly available benchmark datasets CDD, LEVIR-CD, and WHU-CD, the *F*1 scores of MAFF-Net are improved by at least 1%, 2%, and 3%, respectively, compared to other methods. This demonstrates the better performance of our method than other SOTA methods.

However, it should be noted that, as shown in Figure 12, although the proposed model has an advantage in terms of training speed, it cannot be ignored that the number of parameters of the proposed model is relatively large, reaching 49.08 M. This has potential limitations for its practical application in the future. Therefore, in future work, we hope that the network can be made lightweight by using some model compression techniques. In addition, the proposed method solves the CD task of bi-temporal remote sensing images, and in future work, it will focus on the CD task of multi-temporal remote sensing images.

## Figures and Tables

**Table 1:** Summary of contemporary CD methods.

Method	Category	Example Studies
Traditional CD methods	Pixel-based CD	Wang et al. [11], Quarmby et al. [12], Howarth et al. [13], Ludeke et al. [14], Zhang et al. [15], Nielsen et al. [16], Nielsen et al. [17], Bovolo et al. [18], Bovolo et al. [19], Liu et al. [20], Liu et al. [21], Frank et al. [22]
	Object-based CD	Ma et al. [24], Zhang et al. [25], Zhang et al. [26], Gil-Yepes et al. [27], Qin et al. [28]
Deep learning CD methods		FC-EF [38], FC-Siam-conc [38], FC-Siam-diff [38], Daudt et al. [39], FCN-PP [40], BA^2^Net [41], STANet [42], BIT-CD [43], DASNet [44], IFN [45], HDFNet [47]

**Table 2:** Comparison of CDD dataset results. The best scores are highlighted in bold.

Method	*F*1 (%)	*Kappa* (%)	*OA* (%)
CDNet	81.9	79.6	95.9
FC-EF	83.0	80.8	96.0
FC-Siam-conc	84.0	81.9	96.3
FC-Siam-diff	84.8	82.8	96.4
DASNet	90.1	88.7	97.5
IFN	90.6	89.2	97.6
STANet	91.6	90.4	97.9
MAFF-Net	**96.5**	**96.0**	**99.2**

**Table 3:** Comparison of LEVIR-CD dataset results. The best scores are highlighted in bold.

Method	*F*1 (%)	*Kappa* (%)	*OA* (%)
CDNet	78.0	76.9	97.8
FC-EF	80.7	79.7	98.0
FC-Siam-conc	82.2	81.2	98.0
FC-Siam-diff	83.7	82.8	98.3
DASNet	84.6	83.7	98.4
IFN	86.2	85.4	98.6
STANet	86.5	85.9	**98.9**
MAFF-Net	**89.7**	**89.1**	**98.9**

**Table 4:** Comparison of WHU-CD dataset results. The best scores are highlighted in bold.

Method	*F*1 (%)	*Kappa* (%)	*OA* (%)
CDNet	80.4	79.4	98.0
FC-EF	82.3	81.4	98.2
FC-Siam-conc	82.9	82.0	98.2
FC-Siam-diff	83.3	82.5	98.4
DASNet	90.7	90.1	99.0
IFN	88.1	87.5	98.9
STANet	89.8	89.3	99.0
MAFF-Net	**92.4**	**92.1**	**99.4**

**Table 5:** Ablation study of different modules on different datasets. All the scores are described in percentage (%). The best scores are highlighted in bold.

Model					CDD			LEVIR-CD			WHU-CD		
Baseline	FEM	FFM_S1	RRB	FFM_S2	*F*1	*Kappa*	*OA*	*F*1	*Kappa*	*OA*	*F*1	*Kappa*	*OA*
√	×	×	×	×	88.0	86.3	96.9	83.3	82.4	98.2	86.0	85.3	98.8
√	√	×	×	×	93.6	92.7	98.4	87.0	86.3	98.6	89.9	89.3	99.0
√	√	√	×	×	94.6	93.8	98.7	88.2	87.6	98.7	91.4	90.9	99.1
√	√	√	√	×	95.9	95.4	99.0	88.8	88.2	**98.8**	91.9	91.5	99.2
√	√	√	√	√	**96.5**	**96.0**	**99.2**	**89.7**	**89.1**	98.7	**92.4**	**92.1**	**99.4**

## Data Availability

The CDD, LEVIR-CD, WHU-CD datasets are openly available at https://drive.google.com/fifile/d/1GX656JqqOyBi_Ef0w65kDGVto-nHrNs9 (accessed on 1 December 2021), https://justchenhao.github.io/LEVIR/ (accessed on 1 December 2021), http://gpcv.whu.edu.cn/data/building_dataset.html (accessed on 1 December 2021), respectively.

## Abbreviations

The following abbreviations are used in this manuscript:

CD	change detection
HR	high resolution
SOTA	state of the art
VHR	very high resolution
CNN	convolutional neural network
FCN	fully convolutional network
CA	coordinate attention
RRB	refinement residual block
FEM	feature enhancement module
FFM	feature fusion module
GT	ground truth
*OA*	overall accuracy
CD-Net	change detection network
FC-EF	fully convolutional early fusion
FC-Siam-conc	fully convolutional Siamese concatenation
FC-Siam-diff	fully convolutional Siamese difference
DASNet	dual attentive fully convolutional siamese networks
IFN	image fusion network
STANet	a spatial-temporal attention-based method
MAFF-Net	multi-attention guided feature fusion network

## References

[1] Singh A. (1989). Review article digital change detection techniques using remotely-sensed data. Int. J. Remote Sens..

[2] Radke R.J., Andra S., Al-Kofahi O., Roysam B. (2005). Image change detection algorithms: A systematic survey. IEEE Trans. Image Process..

[3] Tison C., Nicolas J.M., Tupin F., Maître H. (2004). A new statistical model for Markovian classification of urban areas in high-resolution SAR images. IEEE Trans. Geosci. Remote Sens..

[4] Papadomanolaki M., Vakalopoulou M., Karantzalos K. (2021). A Deep Multitask Learning Framework Coupling Semantic Segmentation and Fully Convolutional LSTM Networks for Urban Change Detection. IEEE Trans. Geosci. Remote Sens..

[5] Yang J., Weisberg P.J., Bristow N.A. (2012). Landsat remote sensing approaches for monitoring long-term tree cover dynamics in semi-arid woodlands: Comparison of vegetation indices and spectral mixture analysis. Remote Sens. Environ..

[6] Isaienkov K., Yushchuk M., Khramtsov V., Seliverstov O. (2021). Deep Learning for Regular Change Detection in Ukrainian Forest Ecosystem With Sentinel-2. IEEE J. Sel. Top. Appl. Earth Observ. Remote Sens..

[7] Khan S.H., He X., Porikli F., Bennamoun M. (2017). Forest Change Detection in Incomplete Satellite Images with Deep Neural Networks. IEEE Trans. Geosci. Remote Sens..

[8] Sublime J., Kalinicheva E. (2019). Automatic post-disaster damage mapping using deep-learning techniques for change detection: Case study of the Tohoku tsunami. Remote Sens..

[9] Yang X., Hu L., Zhang Y., Li Y. (2021). MRA-SNet: Siamese Networks of Multiscale Residual and Attention for Change Detection in High-Resolution Remote Sensing Images. Remote Sens..

[10] Hussain M., Chen D., Cheng A., Wei H., Stanley D. (2013). Change detection from remotely sensed images: From pixel-based to object-based approaches. ISPRS-J. Photogramm. Remote Sens..

[11] Wang L., Li H. (2011). Soft-change detection in optical satellite images. IEEE Trans. Geosci. Remote Sens. Lett..

[12] Quarmby N.A., Cushnie J.L. (1989). Monitoring urban land cover changes at the urban fringe from SPOT HRV imagery in south-east England. Int. J. Remote Sens..

[13] Howarth P.J., Wickwareg M. (1981). Procedures for change detection using Landsat digital data. Int. J. Remote Sens..

[14] Ludeke A.K., Maggio R.C., Reid L.M. (1990). An analysis of anthropogcnic deforcstation usinglogistic regression and GIS. J. Environ. Manag..

[15] Zhang J., Wang R. (2006). Multi-temporal remote sensing change detection based on independent component analysis. Int. J. Remote Sens..

[16] Nielsen A.A., Conradsen K., Simpson J.J. (1998). Multivariate alteration detection (MAD) and MAF postprocessing in multispectral, bitemporal image data: New approaches to change detection studies. Remote Sens. Environ..

[17] Nielsen A.A. (2007). The Regularized Iteratively Reweighted MAD Method for Change Detection in Multi- and Hyperspectral Data. IEEE Trans. Image Process..

[18] Bovolo F., Bruzzone L. (2007). A theoretical framework for unsupervised change detection based on change vector analysis in the polar domain. IEEE Trans. Geosci. Remote Sens..

[19] Bovolo F., Marchesi S., Member S. (2012). A framework for automatic and unsupervised detection of multiple changes in multitemporal images. IEEE Trans. Geosci. Remote Sens..

[20] Liu S., Bruzzone L., Bovolo F., Du P. (2015). Hierarchical unsupervised change detection in multitemporal hyperspectral images. IEEE Trans. Geosci. Remote Sens..

[21] Liu S., Bruzzone L., Bovolo F., Zanetti M., Du P. (2015). Sequential spectral change vector analysis for iteratively discovering and detecting multiple changes in hyperspectral images. IEEE Trans. Geosci. Remote Sens..

[22] Thonfeld F., Feilhauer H., Braun M., Menz G. (2016). Robust change vector analysis (RCVA) for multi-sensor very high resolution optical satellite data. Int. J. Appl. Earth Obs. Geoinf..

[23] Blaschke T., Hay G.J., Kelly M., Lang S., Hofmann P., Addink E., Feitosa R., Meer F., Werff H., Coillie F. (2014). Geographic object-based image analysis–Towards a new paradigm. ISPRS-J. Photogramm. Remote Sens..

[24] Ma L., Li M., Blaschke T., Ma X., Tiede D., Cheng L., Chen D. (2016). Object-based change detection in urban areas: The effects of segmentation strategy, scale, and feature space on unsupervised methods. Remote Sens..

[25] Zhang Y., Peng D., Huang X. (2017). Object-based change detection for VHR images based on multiscale uncertainty analysis. IEEE Geosci. Remote Sens. Lett..

[26] Zhang C., Li G., Cui W. (2018). High-resolution remote sensing image change detection by statistical-object-based method. IEEE J. Sel. Top. Appl. Earth Observ. Remote Sens..

[27] Gil-Yepes J.L., Ruiz L.A., Recio J.A., Balaguer-Beser Á., Hermosilla T. (2016). Description and validation of a new set of object-based temporal geostatistical features for land-use/land-cover change detection. ISPRS J. Photogramm. Remote Sens..

[28] Qin Y., Niu Z., Chen F., Li B., Ban Y. (2013). Object-based land cover change detection for cross-sensor images. Int. J. Remote Sens..

[29] Tang D., Wei F., Yang N., Zhou M., Liu T., Qin B. Learning sentiment-specific word embedding for twitter sentiment classification. Proceedings of the 52nd Annual Meeting of the Association for Computational Linguistics.

[30] Kim Y., Jernite Y., Sontag D.A., Rush A.M. Character-aware neural language models. Proceedings of the Thirtieth AAAI Conference on Artifcial Intelligence.

[31] Lei T., Zhang Q., Xue D., Chen T., Meng H., Nandi A.K. End-to-end Change Detection Using a Symmetric Fully Convolutional Network for Landslide Mapping. Proceedings of the IEEE International Geoscience and Remote Sensing Symposium (IGARSS).

[32] Li X., Yuan Z., Wang Q. (2019). Unsupervised Deep Noise Modeling for Hyperspectral Image Change Detection. Remote Sens..

[33] Xu Q., Chen K., Zhou G., Sun X. (2021). Change Capsule Network for Optical Remote Sensing Image Change Detection. Remote Sens..

[34] LeCun Y., Bottou L., Bengio Y., Haffner P. (1998). Gradient-based learning applied to document recognition. Proc. IEEE.

[35] He K., Zhang X., Ren S., Sun J. Deep residual learning for image recognition. Proceedings of the IEEE Conference on Computer Vision and Pattern Recognition (CVPR).

[36] Shelhamer E., Long J., Darrell T. (2017). Fully Convolutional Networks for Semantic Segmentation. IEEE Trans. Pattern Anal. Mach. Intell..

[37] Ronneberger O., Fischer P., Brox T. U-net: Convolutional networks for biomedical image segmentation. Proceedings of the International Conference on Medical Image Computing and Computer-Assisted Intervention (MICCAI).

[38] Caye Daudt R., Le Saux B., Boulch A. Fully Convolutional Siamese Networks for Change Detection. Proceedings of the 25th IEEE International Conference on Image Processing (ICIP).

[39] Daudt R.C., Le Saux B., Boulch A., Gousseau Y. (2018). High Resolution Semantic Change Detection. arXiv.

[40] Lei T., Zhang Y., Lv Z., Li S., Liu S., Nandi A.K. (2019). Landslide Inventory Mapping from Bi-temporal Images Using Deep Convolutional Neural Networks. IEEE Geosci. Remote Sens. Lett..

[41] Zhang Y., Zhang S., Li Y., Zhang Y. (2020). Coarse-to-Fine Satellite Images Change Detection Framework via Boundary-Aware Attentive Network. Sensors.

[42] Chen H., Shi Z. (2020). A Spatial-Temporal Attention-Based Method and a New Dataset for Remote Sensing Image Change Detection. Remote Sens..

[43] Chen H., Qi Z., Shi Z. (2021). Remote Sensing Image Change Detection With Transformers. IEEE Trans. Geosci. Remote Sens..

[44] Chen J., Yuan Z., Peng J., Chen L., Huang H., Zhu J., Liu Y., Li H. (2020). DASNet: Dual attentive fully convolutional siamese networks for change detection of high resolution satellite images. IEEE J. Sel. Top. Appl. Earth Obs. Remote Sens..

[45] Zhang C., Yue P., Tapete D., Jiang L., Shangguan B., Huang L., Liu G. (2020). A deeply supervised image fusion network for change detection in high resolution bi-temporal remote sensing images. ISPRS J. Photogramm. Remote Sens..

[46] Hou Q., Zhou D., Feng J. Coordinate Attention for Efficient Mobile Network Design. Proceedings of the IEEE Conference on Computer Vision and Pattern Recognition (CVPR), Virtually.

[47] Zhang Y., Fu L., Li Y., Zhang Y. (2021). HDFNet: Hierarchical Dynamic Fusion Network for Change Detection in Optical Aerial Images. Remote Sens..

[48] Lin M., Chen Q., Yan S. Network in network. Proceedings of the International Conference on Learning Representations (ICLR).

[49] Szegedy C., Liu W., Jia Y., Sermanet P., Rabinovich A. Going deeper with convolutions. Proceedings of the IEEE Conference on Computer Vision and Pattern Recognition (CVPR).

[50] Yang L., Chen Y., Song S., Li F., Huang G. (2021). Deep Siamese Networks Based Change Detection with Remote Sensing Images. Remote Sens..

[51] Wang D., Chen X., Jiang M., Du S., Xu B., Wang J. (2021). ADS-Net:An Attention-Based deeply supervised network for remote sensing image change detection. Int. J. Appl. Earth Obs. Geoinf..

[52] Zeiler M.D., Krishnan D., Taylor G.W., Fergus R. Deconvolutional networks. Proceedings of the IEEE Conference on Computer Vision and Pattern Recognition (CVPR).

[53] Dumoulin V., Visin F. (2016). A guide to convolution arithmetic for deep learning. arXiv.

[54] Augustus O., Vincent D., Chris O. (2016). Deconvolution and Checkerboard Artifacts. Distill.

[55] Woo S., Park J., Lee J.Y., So Kweon I. Cbam: Convolutional block attention module. Proceedings of the European Conference on Computer Vision (ECCV).

[56] Ioffe S., Szegedy C. Batch Normalization: Accelerating Deep Network Training by Reducing Internal Covariate Shift. Proceedings of the International Conference on Machine Learning (ICML).

[57] Glorot X., Bordes A., Bengio Y. Deep sparse rectifier neural networks. Proceedings of the International Conference on Artificial Intelligence and Statistics (AISTATS).

[58] Gulcehre C., Moczulski M., Denil M., Bengio Y. Noisy activation functions. Proceedings of the International Conference on Machine Learning (ICML).

[59] Yu C., Wang J., Peng C., Gao C., Yu G., Sang N. Learning a discriminative feature network for semantic segmentation. Proceedings of the IEEE Conference on Computer Vision and Pattern Recognition (CVPR).

[60] Lebedev M., Vizilter Y.V., Vygolov O., Knyaz V., Rubis A.Y. (2018). Change Detection in Remote Sensing Images Using Conditional Adversarial Networks. Int. Arch. Photogram. Remote Sens. Spat. Inf. Sci..

[61] Ji S., Wei S., Lu M. (2018). Fully convolutional networks for multisource building extraction from an open aerial and satellite imagery data set. IEEE Trans. Geosci. Remote Sens..

[62] Alcantarilla P.F., Simon S., Germán R., Roberto A., Riccardo G. (2018). Street-view change detection with deconvolutional networks. Auton. Robot..

[63] Li H., Kadav A., Durdanovic I., Samet H., Graf H.P. (2016). Pruning filters for efficient convnets. arXiv.

[64] Vadera M.P., Marlin B.M. (2021). Challenges and Opportunities in Approximate Bayesian Deep Learning for Intelligent IoT Systems. arXiv.

